# 

**Published:** 1891-06-06

**Authors:** 


### NOTICE TO CORRESPONDENTS.

All MS., Letters, Books for Review, and other matters intended for the Editor should be addressed The Editor, The Lodge, Porchester Square, London, W.

The Editor cannot undertake to return rejected M.S., even when accompanied by stamped directed envelope.

### Notice to Advertisers and Subscribers.

All Advertisements, Orders for Copies of The Hospital, and Business Communications should be addressed to The Publisher, *not to the Editor*, The Hospital, 140, Strand, W.C.

The Cost of Subscription, post free, is as follows:—Per week, 1½d.; per quarter, 1s. 8d.; per half-year, 3s. 3d.; per annum, 6s. 6d. Foreign:—Per annum, 8s. 8d.; payable, in all cases, in advance.

NOTICE.—The Index for Vol. IX.. (ending March, 1891) is on sale at the Office of this Journal, price 2½d., post free.

### General Charities.

[This Column is devoted to an account of work of charitable institutions other than Medical Charities. The Editor will be glad to receive reports or news of work at 140, Strand. *Philanthropy* should be plainly written on the envelope.]

The Committee of the Football Charity Fund have sent £29 to the **LONDON PLAYING FIELDS COMMITTEE.**

The hearts of the children in the Irish schools in London of the **BENEVOLENT SOCIETY OF ST. PATRICK** have been gladdened by the receipt from Mr. George Findlay, on behalf of the London and North-Western Railway Company, of a series of photographs of Irish scenery. The series, which is of no small value for educational purposes, consists of a number of artistic views chosen from many taken from time to time for the Company.

**THE NATIONAL PHYSICAL RECREATION SOCIETY** does in gymnastics and jumping what the London Playing Fields' Committee is endeavouring to do in cricket. It was founded for the purpose of assisting the working classes to obtain physical recreation, and has already established and furnished with honorary instructors over 200 classes composed of working people who are unable to pay for professional gymnastic tuition. From the gymnasium attached to Exeter Hall, where the annual competition takes place, they have already sent out no fewer than 80 such qualified instructors.

A General Court of the **SCHOOL FOR THE INDIGENT BLIND**, St. George's Fields, Southwark, of which there is a junior branch at Linden Lodge, Wandsworth Common, will be held at the Cannon Street Hotel on Thursday, June 11th. The Secretary wishes to remind the public that all kinds of baskets, plain and fancy door mats, worsted rugs, cocoanut matting, sash and clothes lines, knitting, &c., manufactured by the inmates, are sold at the School at moderate prices, and that the purchase of any of them will materially assist the charity.

Admiral Sir F. Leopold McClintock appeals for support for the **ROYAL ALFRED AGED MERCHANT SEAMEN'S INSTITUTION**, in order to increase its power for good. He states that one friend offers £500 for the endowment fund provided that by the close of this year the sum of £4,500 has been collected. Though the Committee hope to get a considerable amount towards this end at the approaching festival dinner, they look to the outside help of the public as well. Those who visit the Naval Exhibition will on all sides be reminded of the perils of the sea, and of our entire dependence upon our merchant seamen for most of the luxuries and comforts, and even the necessaries of life, which surround us. The institution is essentially a national one, and we venture to think that the public only requires to have its attention drawn to its pressing needs to ensure a generous response to the earnest appeal made for our worn-out merchant seamen, so often left destitute and friendless, and this after the life of hardship, privation, and danger inseparable from a sailor's career. Donations sent to 58, Fenchurch Street, E.C., will be most gratefully acknowledged.

The hardships of a sailor's life do not consist solely of the privations and dangers with which he has to contend when at sea. At the best his life is monotonous in the extreme. Even if he has a family and a home he is rarely able to enjoy them, for his life is destined to be apart from them. When he endeavours to bring some change into the dreary routine of sea life by landing at a port at which his vessel touches, the moment he reaches the shore he is surrounded by every temptation, of the vilest description, placed in his path by designing scoundrels who thrive on his simplicity and frankness. Experience has long shown that there is but one way of counteracting these evils, and that is by establishing in every port a sailors' home, where clean accommodation and genial company are to be found. With this view in sight, the **BRITISH AND FOREIGN SAILORS' SOCIETY** was founded at the commencement of the century. The Sailors' Institute in Mercer Street, Shadwell, which, like many others, has been established in connection with this Society, has been an untold boon to thousands of men, who, in place of a rum shop reeking with foul language and fouler air, find a decent home where they can read a paper, write their letters, and enjoy every advantage of a comfortable club. The progressiveness of the Society is shown by the fact that they have recently extended their operations to ports in South America, and there are now connected with it seventy-nine stations in seventy-five ports, and sixty-four institutes, rests, homes, and reading-rooms on shore. But at the present moment the Society is anxious to provide for the needs of sailors nearer home. At the Milwall Docks, situated in the poor district of the Isle of Dogs, there is no provision for them, though it is hoped that the much-needed institute will very shortly become an established fact. The Dock Company has granted on reasonable terms a vacant plot at the entrance to the Dock, and a lady has become resposible for £1,200 towards carrying out the project, which requires another £1,000 to complete it. Donations for this purpose and the general work of the Society will be gratefully acknowledged by Mr. Edward W. Matthews, the Secretary, Mercer Street, Shadwell, E.

### Scraps and Gleanings.

A BAZAAR is being held this week in aid of the Queen's Jubilee Hospital at Kensington.

THE Princess of Wales will open the new Children's Hospital at Eastbourne on June 20th.

THE Duke of Westminster has become an annual subscriber of £500 to the London City Mission.

THE London Physical Society held its last meeting at Cambridge, when some interesting papers were read.

THE Goldsmiths' Company have made a grant of £50 in aid of the funds of the Royal Westminster Ophthalmic Hospital.

THE Grocers' Company have sent £100 to the Hon. Alfred Lyttelton, the Treasurer of the Children's Country Holidays Fund.

THE Governors of St. George's Hospital have decided to spend £30,000 on additions, alterations, and improvements to the building.

THE St. John Ambulance first aid certificate has been won by 128 men connected with the Great Northern Railway during the last year.

THE Kensington Philanthropic Society held their annual dinner on May 26th. The Earl of Aberdeen presided, and subscriptions amounting to £200 were announced.

DR. KOCH is engaged in separating the efficient substance contained in tuberculine, so that its qualities may be chemically determined, as in the case of other medicaments.

ON May 23rd, the Duchess of Teck opened an exhibition at Morley Hall, Regent Street, the profits on which are to be devoted to the Mildmay Mission Hospital at Bethnal Green.

THE small Queen of Holland made her first speech when laying the foundation stone of a hospital. In a sweet, childish voice she gravely hoped "the building would prove a blessing."

A PATIENT at the Queen's Hospital, Birmingham, was in the lift with an attendant when some hitch in the working caused the cage to descend with a rush, and the patient was shot out and severely injured.

IN recognition of the munificence of the late Andrew Pickard, Esq., of Ossett, who bequeathed the sum of £1,000 to the Dewsbury and District Infirmary, the Board of that institution have resolved to name one of the infirmary wards the "Pickard Ward."

DR. GRIFFITH, of the Zenana Medical College, asks us to announce that in connection with the Hospital for Women and Children he is forming a dispensing and midwifery class, &c., for men who are preparing to be medical missionaries, but who cannot afford fees.

THE jubilee dinner of the Pharmaceutical Society of Great Britain took place at the Criterion Restaurant on May 27th. Mr. M. Carteighe, President, was in the chair, and the large gathering included Sir Lyon Playfair, Sir Joseph Lister, Sir Dyce Duckworth, Dr. J. W. Black, and Mr. T. Bryant.

THE Committee of the Football Charity Fund have forwarded £20 to each of the following institutions: North-Eastern Hospital for Children, St. Mary's Institute (East Greenwich), Blackheath and Charlton Cottage Hospital, Metropolitan and City Police Orphanage, and London Playing Fields Committee.

FUNDS are needed by the Victoria Cottage Hospital, Barnet, in order to provide a proper kitchen, accident ward, accommodation for isolating patients, and suitable sleeping accommodation for the staff. The amount required to provide these is £850, of which £150 is in hand and £200 promised, leaving £500 still to be raised.

THE returns presented at the last meeting of the Asylums Board, showed that during the fortnight ending May 28th, there were 1,128 patients in the hospitals. These included 876 cases of scarlet fever, 149 of diphtheria, 3 of typhus fever, and 78 of enteric fever. The total shows a decrease of 31 on that for the preceding fortnight.

THE Commander-in-Chief has approved of the Volunteer Medical Staff Corps of London, Edinburgh, Aberdeen, and Norwich being permitted to encamp at Aldershot in the first week of August, for training with the regular Medical Staff Corps; and for the Leeds Corps at the same time to go to Netley Hospital for the same purpose.

THE annual meeting of the Infant Orphan Asylum, Wanstead, was held on May 28th at the Cannon Street Hotel, Mr. W. Evill in the chair. The report stated that 620 children had been benefited by the asylum during 1890, and there had been only one death. After the adoption of the report, the poll was opened for the election of 15 boys and 15 girls.

AT a special meeting of the Charity Organization Society, a paper on "The Care of the Feeble-Minded and Epileptic" was read by Miss Stacey, a Birmingham guardian, and a resolution was carried in favour of Boards of Guardians maintaining the feeble-minded and epileptic in the same way as the deaf, dumb, and blind are provided for by Parliament.

A VERY largely-attended meeting was held in Oxford on the 29th ult., in support of Lady Dufferin's Fund for supplying female medical aid to the women of India. Speeches were made by Lord Northbrook, Lord Reay, Sir Henry Acland, Lady Dufferin, and others. Sir Harry Verney read a few lines he had received from Miss Florence Nightingale, commending the movement to public support.

THE payments made by the Metropolitan Asylums Board during 1890 for the land hospital ambulance services were £8,095 4s. 5d., and for the river services £3,824 3s. 7d., the total making £11,919 8s. The land services cost 18 per cent. in excess of the 1889 amount, though the work done showed an excess of 75 per cent. In addition to the conveyance of patients to hospital, much work was entailed by transfer of convalescents and discharged cases, the vehicles in all having traversed during the year no fewer than 83,061 miles.

### THE STUDENT OF MEDICINE.

[All communications for this Supplement should be addressed to THE EDITOR of THE HOSPITAL, at 140, Strand, with the words, "Students' Column," written in the left hand top corner of the envelope.]

**THE UNITED HOSPITALS ATHLETIC CLUB.**

The United Hospitals Athletic Club having lately become more prominent, not only among the hospitals, but in the athletic world at large, a brief account of its origin, somewhat chequered career, and present condition may be of interest. This club was inaugurated in the year 1867, and is therefore considerably the oldest of the United Hospitals clubs. The first President was Sir W. Ferguson, and it may here be said that the list of Presidents is indeed a roll of the most eminent medical celebrities of our times, including such names as Jenner, Paget, Gull, Prescot Hewett, Andrew Clarke, Lister, &c. The club immediately became a great success, and its meetings were extremely popular and well-attended, not only by the students and their friends, but also by the general public. In addition to the sports, the club at this time held swimming races, but these were never well supported, and were soon discontinued. An annual dinner was also held after the sports for some years, and was usually a success. This flourishing condition lasted for more than twelve years, when, owing to many of the hospitals holding their own athletic meetings, and, perhaps it must be admitted, partly to a certain amount of careless management, a gradual deterioration set in. This is well shown by the balance-sheet. In 1883 there was a deficit of £12, in 1884 it had risen to £29, in 1885 to £39, and reached its culminating point in 1886, when it was returned at £60. The club had now reached its lowest ebb, and it was in this year that a determined effort was made by the Secretary and Treasurer, A. E. Nuttall and J. Errington Ker, to raise it once more to its former position. The debt on the club was reduced in 1888 to £15, and the meetings of 1886 and 1887 were markedly successful, but it was not until 1888 that the club may be said to have regained its original flourishing condition. W. Kent-Hughes became Secretary, and the present position of the club is undoubtedly in a great measure due to his efforts, ably seconded by his brother officers. Two important innovations were now introduced, namely, the substitution of medals for prizes and the institution of matches against Edinburgh, Cambridge, and the L.A.C. With regard to the former, they were thought more suitable to the t ne of the meeting, besides—a very important consideration—being less expensive when once the original cost of the die had been defrayed. The medals, although somewhat large, are very neatly designed, having on one side the arms of all the hospitals, and compare favourably with those given by the Universities and other clubs. The matches were undertaken not only for the sake of the U.H.A.C., but also in the cause of amateur athletics generally; for it was felt that if the better-class clubs did not take some steps to promote more meetings for genuine sport, the professional-amateurism and pot-hunting, which had unfortunately become almost synonymous with amateur athletics, would completely destroy this valuable pastime. The first match was against Edinburgh in July, 1889, when the U.H.A.C. won by 7 points to 3. In the second match, against Cambridge, in March of 1890, the U.H.A.C. only managed to secure one event, but it is only fair to add that, whilst there was scarcely a man in their team fit, the Cambridge team was in splendid form, defeating Oxford by almost as large a majority very shortly after. On July 1st the U.H.A.C. again defeated Edinburgh by 9 points to 1, and on the 5th met the L.A.C., when the latter, with a very powerful team, proved victorious by 7 points to 4, after, perhaps, the best display of form seen in London this season. The U.H.A.C. team last year was an exceptionally strong one, including such well-known athletes as Kent-Hughes, Marshall, B. C. Green, Hogarth (the amateur champion broad-jumper), and Quennell, whilst among the recent additions are Wace, Fraser, and Pierre, all of whom have proved themselves capable of high-class performances. In 1888 there was a deficit of about £15, but at the general meeting this year the treasurer was able to announce a balance of about the same amount, in spite of the fact that the die alluded to above had to be purchased, involving an extra expenditure of £35. Thus by careful management, and collecting a few extra subscriptions, £65 had been saved on the working expenses of the club in two years. In conclusion, it is hardly necessary to point out that, although the club has, through the efforts of a few, been brought once more into a flourishing state,

### THE STUDENT OF MEDICINE—(*continued*).

it can only be maintained in that condition by the united support of the general body of students, past and present.

### APPOINTMENTS.

At the Chelsea Hospital for Women, Fulham Road, J. A. Shaw-Mackenzie, M.B., M.R.C.S., has been appointed pathologist. At the East London Hospital for Children John W. MacVine, M.B., C.M., has been appointed house physician. At the Moorfields Ophthalmic Hospital A. Stanford Morton, M.B., F.R.C.S.Eng., has been appointed assistant house surgeon. At the North-West London Hospital W. H. Vickery, M.R.C.S., L.R.C.P., has been appointed assistant resident medical officer. At the Birkenhead Lying-in Hospital Alice Ker, M.D., has been appointed honorary surgeon. At the City of London Lunatic Asylum, Stone, near Dartford, William G. Evans, F.R.C.S.Eng., L.R.C.P.Lond., has been appointed clinical assistant. At the City of Newcastle Asylum, Gosforth, Robert G. Smith, M.A., B.Sc., M.R.C.S., has been appointed assistant medical officer. At the Devonshire Hospital, Buxton, F. J. Salter, L.R.C.P., L.R.C.S.Edin., has been appointed assistant house surgeon. At the Birmingham and Midland Free Hospital for Sick Children, George William Powell, B.A., M.B., B.Ch., has been appointed extra acting physician. At the Holloway Sanatorium, Virginia Water, Jane B. Henderson, L.R.C.P., L.R.C.S.Edin., has been appointed third assistant medical officer. At the Ida Hospital General Infirmary, Leeds, Algernon Wear, M.B., B.S.Durh., has been appointed resident medical officer. At the Manchester Southern Hospital for Women and Children J. Harold Bailey, M.B., Ch.B.Vict., has been appointed house surgeon. At the North Brierley Union, Thomas Logan, M.D.Aberd., has been appointed medical officer. At Oxford University W. D. Halliburton, M.D., B.Sc., M.R.C.S., has been appointed examiner in physiology. At the Royal South London Ophthalmic Hospital, St. George's Circus, L. Vernon Cargill, has been appointed clinical assistant. At the Royal Portsmouth, Portsea, and Gosport Hospital, E. T. Crouch, M.R.C.S., L.S.A., has been appointed honorary surgeon. At the Wallasey Dispensary Gilbert H. Griffiths, M.R.C.S., L.R.C.P.Lond., has been appointed house surgeon. At King's College Hospital, H. S. Ballance, M.R.C.S., L.R.C.P., J. J. N. Morris, M.R.C.S., L.R.C.P., and H. S. Sandifer, M.R.C.S., L.R.C.P., have been appointed house surgeons; and J. J. Waddelow, L.S.A., assistant house accoucheur. At St. Thomas's Hospital, C. R. Boz, B.Sc.Lond., L.R.C.P., M.R.C.S., and T. H. Kellock, M.A., M.B., B.C.Cantab., L.R.C.P., F.R.C.S., house physicians; S. G. Toller, L.R.C.P., M.R.C.S., W. S. Griffiths, M.A., M.B., B.C.Cantab., L.R.C.P.. M.R.C.S., W. G. G. Stokes, B.A., M.B., B.C.Cantab., L.R.C.P., M.R.C.S., and L. A. J. Rouillard, M.B., B.C.Cantab., L.R.C.P., M.R.C.S., house surgeons; L. Cobbett, L.R.C.P., F.R.C.S., and C. Wyman, M.A., M.B., B.C.Cantab., L.R.C.P., M.R.C.S., assistant house surgeons; C. R. F. Stillwell, L.R.C.P., M.R.C.S., L.S.A., and H. Low, M.A., M.B., B.C.Camb., L.R.C.P., M.R.C.S., non-resident house physicians; J. R. Harper, L.R.C.P., M.R.C.S., resident accoucheur; W. F. Umney, L.R.C.P., M.R.C.S., senior obstetric clerk; F. E. Forward, F.R.C.S., L.R.C.P., and C. H. Usher, B.A., M.B., B.C.Cantab., non-resident ophthalmic house surgeons; F. W. Beville, L.R.C.P., M.R.C.S., and A. E. Price, L.R.C.P., M.R.C.S., clinical assistant to the skin department; P. J. Atkey, L.R.C.P., M.R.C.S., and T. A. M. Forde, L.R.C.P., M.R.C.S., clinical assistant to the throat department. At the Aberdeen Sick Children's Hospital, Alexander Mackay, M.A., M.B., C.M., has been appointed resident surgeon. At the Birmingham City Asylum, George A. Watson, M.B., C.M.Edin., has been appointed assistant medical officer, and Michael Grabham, M.A., M.B., B.Sc.Cantab., resident clinical assistant. At the Great Yarmouth Hospital, George Walker, M.R.C.S., L.R.C.P., has been appointed resident house surgeon. At the Jenny Lind Infirmary for Sick Children, Norwich, F. W. Burton-Fanning, M.B. Cantab., &c., has been appointed physician. At the Royal Infirmary, Newcastle-on-Tyne, W. D. Arnison, M.D., Dunelm, has been appointed senior house physician. At the Sussex County Asylum, W. D. Calvert, L.R.C.P., M.R.C.S., has been appointed house surgeon.

### VACANCIES.

The following vacancies are announced this week, the amount of salary in each case being given after the name of the institution:

**Resident Medical Officers.**

County Asylum, Whittingham, Preston, Lancashire, Assistant Medical Officer and Pathologist.—Salary £200, with board, etc.

### THE STUDENT OF MEDICINE—(*continued*).

Halifax Infirmary and Dispensary, Assistant House Surgeon—Salary £50, &c.
Kidderminster Infirmary, House Surgeon—Salary £140, &c.
Southport Infirmary, Assistant to the House Surgeon—Board, &c.
Worcester Amalgamated Friendly Societies' Medical Association—Assistant Medical Officer—Salary £140, &c.
Bristol City Lunatic Asylum, Second Assistant Medical Officer—Salary £120, &c.
Derbyshire General Infirmary, Resident Assistant House Surgeon.
Royal London Ophthalmic Hospital, Moorfields, E.C., Junior House Surgeon—Salary £50, &c.
St. Thomas's Hospital, Resident Assistant Surgeon.
St. Mary's Hospital, Dental Surgeon.
West London Hospital, Hammersmith Road, House Physician and House Surgeon.

#### Non-Resident Medical Officers.

University of Glasgow, Assistant Examiner in Physiology—Annual fee £30.
Hull Royal Infirmary, Honorary Ophthalmic Surgeon and four Honorary Assistant Surgeons.
Queen's College, Birmingham, Medical Tutor and Demonstrator of Anatomy, also Demonstrator of Anatomy.

### NOTES FROM THE SCHOOLS.

#### Cambridge.

The University having been deprived of its High Steward by the death of Earl Powis, Lord Walsingham has been appointed to the post.

Although the May Week is still some way off, rumours of several entertainments are getting about, among which we hear of a pastoral play in Downing, "Much Ado About Nothing," and "Love in a Mist."

Pembroke College has once more brought itself to the notice of the public. According to current accounts it has refused to admit a student because he was a Roman Catholic, although he undertook to "oblige the authorities" by attending chapel, as the other men are compelled to do. It is not long ago we heard that the Master of this otherwise excellent College, refused to admit a student on the grounds that he was a science man. In fact, we are given to understand that no science or medical students are admitted now at all, because the "Dons" of the College do not, and will not, understand the complexity of their examinations.

#### Oxford.

It was a deplorably wet Eights Week at the University, and cousins and sisters spoilt no end of smart frocks, and caught a fearful number of colds. The boats themselves have also not presented much of interest. The first four boats closed up somewhat at the start, but that was all. Pembroke have, however, been enabled to carry the traditional rose fixed in their stern, having gone up five places in the four nights, from thirteenth to eighth.

A publication called the *New Rattle* has been issued, but of its contents and general literary "style" the less said the better. Another one, the *Clown*, has also just appeared, which is distinctly an advance on the former.

### SCHOLARSHIPS AND PRIZES.

#### St. Mungo's College, Glasgow.

FACULTY OF MEDICINE.

PRIZE LIST—WINTER SESSION, 1890-1.

SURGERY (Professor: David N. Knox, M.A., M.B.).—*Gold Medal*, W. P. Willis; *Honorary Certificates* (in order of merit): J. C. Beckett, J. Baldwin, J. Hodgson, J. P. T. Burke, H. C. Darby, S. Firth, H. B. Ord.

MATERIA MEDICA (Professor: John Dougall, M.D.).—*Prize*, W. Crewdson; *Certificates of Honour* (in order of merit): J. G. McColl, D. Stephen, R. T. Jones, J. W. Lax, R. H. Manson, H. P. Weston.

MEDICINE (Professor: Alexander Robertson, M.D.).—*First Prize*, J. Hodgson; *Second Prize*, H. B. Ord; *Honorary Certificates* (in order of merit): Miss Ina Cadell, R. T. Davies, J. C. Beckett, S. Firth, W. P. Willis.

CLINICAL MEDICINE (Professor: David C. McVail, M.B.).—*Gold Medal* (presented by D. C. McVail, M.B.), R. T. Davies; *Certificates of Honour*: D. McCrorie, J. Wardrop.

PATHOLOGY (Lecturer: J. Lindsey Steven, M.D.).—*First Prize*, W. P. Willis; *Honorary Certificates* (in order of merit): W. Melville, J. Devon, R. T. Davies, I. Baldwin. *Prize* for best report of Lectures and Demonstrations, Miss Ina Cadell.

CHEMISTRY (Professor: James M. Milne, Ph.D., F.I.C.).—*First Prize*, W. J. Owen; *Second Prize*, E. H. Hilton; *Honorary Certificates* (in order of merit), 1, L. A. Williams, 2, H. Roberts, 3, J. H. Robinson, 4, H. McKay, 5, J. McLaren, 6, S. Brooks. *Chemical Section*: J. Morrison (*Prize*).

ANATOMY (Professor: Henry E. Clark, M.R.C.S. Demonstrator: Henry M. Borland, M.B., C.M.).—*Senior Division*—*Gold Medal* (presented by Alex. Macdonald, Esq., L.R.C.P.), A. W. Hall; *Silver Medal* (presen-

THE STUDENT OF MEDICINE—*(continued)*.

ted by W. Mackenzie, Esq., L.R.C.P.), W. A. Reidy; *Honorary Certificates* (in order of merit), J. G. McColl and J. Livingstone, æq., J. Willett, T. W. Bartlett, W. Crewdson. *Junior Division—Gold Medal* (presented by J. John Macintyre, Esq., M.D.), H. McKay; *Silver Medal* (presented by W. Mackenz'e, Esq., L.R.C.P.), J. H. Robinson; *Honorary Certificates* (in order of merit), E. Allen, S. C. Brooks, E. H. Roberts, J. Tibbetts, J. McLean and C. A. Bois, æq.

PRACTICAL ANATOMY.—*Senior Division—Honorary Certifica'es*, T. W. Bartlett, W. Crewdson, R. T. Jones, J. Livingstone, R. Martin, R. H. Manson, J. Munro, J. E Proud, W. A. Reidy, D. Stephen, J. Willet. *Junior Division*—E. Allen, C. A. Bois, S. C. Brooks, E. H. Hilton, H. McKay, J. McLean, W. J. Owen, E. H. Roberts, J. H. Robinson, J. Tibbetts. *Class Prosector*—John Willet. Special prize for the highest marks in the written examinations, Senior Anatomy Class, C. Rutherford.

NATURAL HISTORY (Professor: E. E. Prince, B.A., F.L.S.).—*Systematic Class—Silver Medal and Frst Certificate*, L. A. Williams; *Second Certificate*, A. Robertson.

PRACTICAL ZOOLOGY.—*First Certificate*, A. Robertson; *Second Certificate*, W. Hanson.

PHYSIOLOGY (Professor: John Barlow, M.D., F.R.C.S.).—*Gold Medal*, T.W.Bartlett; *Honorary Certificates* (in order of merit), R. H. F. Bostock, D. Stephen, J. Livingstone, W. A. Reilly, W. Crewdson.

### ATHLETICS.

### Rowing.

*United Hospitals Rowing Club.*—A meeting of the above club was held on Tuesday, May 19th, and it was decided to hold the annual inter-hospital race for the challenge cup on June 26th (heats) and June 27th (final).

*The Middlesex Hospital Rowing C'ub.*—This club held their annual scratch fours on Tuesday, May 12th, when the winning crew was: R. S. Thomas (bow), T. H. Wilson (2), H. S. Coven (3), C. H. Nicholson (stroke), and H. C. Poiteous (cox.)

### Cricket,—SATURDAY, MAY 23RD.

*St. Thomas' Hospital v. Richmond.*—This match was played at Richmond, with the result of a win for the hospital by 114 runs. Scores: St. Thomas' Hospital, 160; Richmond, 46 and 67 for seven wickets.

*London Hospital v. Surbiton.*—This was played at Surbiton on a very unsatisfactory and sticky wicket, with the result that Surbiton won. Scores: London Hospital, 46 and 94; Surbiton, 63.

*Guy's Hospital v. Crystal Palace.*—This match was played on the Palace grounds, and the hospital won in the first innings by 138 runs.

EDINBURGH, MAY 23RD.

*University v. Royal High School.*—Royal High School won by 92 to 70. F. W. Menzies made 15 for the Varsity.

*The University Australasians* were very successful in beating the *West of Scotland* by 130 to 103. R. Maxwell (Australasian) was not out for 60, and "J. Robertson" (W. of S.), 35.

MAY 29TH.

*St. Bartholomew's Hospital v. Maidenhead.*—This match was played on the ground of the latter, at Maidenhead, in beautiful weather, and resulted in a win for Maidenhead by 22 runs. Scores: First innings, Bartholomew's, 70; Maidenhead, 92. Second innings, Bartholomew's, 110 for three wickets.

*London Hospital v. Buckhurst Hill.*—This match was played at Buckhurst Hill, and the Hospital was defeated by 66 runs. Webb scored 46 for the Hospital, and McEwen 75 for Buckhurst Hill. Scores: London Hospital, 109; Buckhurst Hill, 175.

### Edinburgh University Cycling Club.

May 9th.—Six members to Kinross, viâ Granton and Burntisland. May 16th.—Annual Fête at Peebles.

### EVENTS FOR THE MONTH OF JUNE.

### Cricket Matches.

GUY'S HOSPITAL.—First Eleven.—June 6th, v. Brentwood, at Brentwood; 10th, v. Streatham, at Streatham; 13th, v. Kensington Park, at Kensington; 16th, v. Blackheath, at Rectory Field; 20th, v. Surbiton, at Surbiton; 27th, v. Rickmansworth, at Rickmansworth. Second Eleven.—June 6th, v. King's College School, at School; 10th, R.M.A., at Woolwich; 13th, v. Forest School, at School; 20th, v. Highgate School, at School; 27th, v. Wimbledon School, at School.

LONDON HOSPITAL.—June 3rd, v. H.A. Company, at Finsbury Square; 6th, v. Willesden, at Willesden; 10th, v. Royal Naval School, at Eltham; 13th, v. Leys School, at Cambridge; 20th, v. Ware, at Ware; 24th, v. Blackheath School at Blackheath; 27th, Witham, at Witham.

ST. MARY'S HOSPITAL.—June 6th, v. Maidenhead, at Maidenhead; 10th, v. Hendon, at Hendon; 11th, v. Bank of England, at Catford Bridge; 13th, v. Leavesden Asylum, at Leavesden; 17th, v. Farnham, at Farnham; 27th, v. Cane Hill, at Cane Hill. Second Eleven.—20th, v. St. Mark's, at Windsor; 27th, v. King's College, at Wormwood Scrubbs.

MIDDLESEX HOSPITAL.—June 1st, Cup Tie, v. (winner of Guy's and Barts.), at Richmond; 6th, v. Cane Hill Asylum, at Purley; 13th, v. Epsom College, at Epsom; 17th, v. The Philberds, at Holyport; 20th, v. Brentwood, at Brentwood; 24th, v. Ware Town, at Ware; 27th, v. High Wycombe, at Wycombe.

### Student's Medical Societies.

UNIVERSITY COLLEGE MEDICAL SOCIETY.—June 10th, annual general meeting. Address by the President. Chairman, the President.

WESTMINSTER HOSPITAL MEDICAL SCHOOL GUTHRIE SOCIETY.—June 11th.

